# Supplementary material for: An open-source framework for large-scale, flexible evaluation of biomedical text mining systems
Source: J Biomed Discov Collab. 2008 Jan 29;3:1. doi: 10.1186/1747-5333-3-1 (PMC2276192; doi:10.1186/1747-5333-3-1)
Supplement: Additional file 1 — Evaluation platform source code. The additional file contains the software evaluation framework discussed in this paper. The most current release can be downloaded from SourceForge.net [1]. See the accompanying README file for instructions on installation and use. Also included in the distribution is a collection of UIMA wrappers for some commonly used BioNLP tools and annotated corpora. [file 1747-5333-3-1-S1.zip › bionlp_uima_repository-1.0.1/docs/api/edu/uchsc/ccp/util/nlp/stats/NLPStats.html]

NLPStats (Center for Computational Pharmacology Util/UIMA API)


|  |  |  |
| --- | --- | --- |
| **Overview** \| **Package** \| **Class** \| **Tree** \| **Deprecated** \| **Index** \| **Help** | |  |
| PREV CLASS   **NEXT CLASS** | **FRAMES**    **NO FRAMES**     **All Classes** |  |
| SUMMARY: NESTED \| FIELD \| CONSTR \| METHOD | DETAIL: FIELD \| CONSTR \| METHOD |  |


---


## edu.uchsc.ccp.util.nlp.stats Class NLPStats

```
java.lang.Object
  edu.uchsc.ccp.util.nlp.stats.NLPStats
```

---

``` public class NLPStats extends Object ```

This class implements some commonly used statistics in the field of NLP, namely precision, recall, F-measure, and the Dice coefficient.

For a give count of true positives (TP), false positives (FP), and false negatives (FN), precision (P), recall (R), and F-measure (F) are computed
as:

P = TP/(TP+FP)

R = TP/(TP+FN)

F = 2PR/(P+R)

**Author:**
:   William A Baumgartner, Jr.

---

**Constructor Summary**

`NLPStats()`


| **Method Summary** | |
| --- | --- |
| `static double` | `computeClassicDice(Set<String> aTokens, Set<String> bTokens)`             Computes the Dice Coefficient as described in Hersh et al. |
| `static double` | `computeClassicDice(String aString, String bString)`             Computes the Dice Coefficient as described in Hersh et al. |
| `static double` | `computeFMeasure(double precision, double recall)`             Given precision and recall, compute the F-measure |
| `static double` | `computeFMeasure(int tp, int fp, int fn)`             Given counts of TPs, FPs, and FNs, compute the F-measure |
| `static double` | `computePrecision(int tp, int fp)`             Given counts of TPs and FPs, compute the precision |
| `static double` | `computeRecall(int tp, int fn)`             Given counts of TPs and FNs, compute the recall |

**Methods inherited from class java.lang.Object**

`clone, equals, finalize, getClass, hashCode, notify, notifyAll, toString, wait, wait, wait`

**Constructor Detail**

### NLPStats

```
public NLPStats()
```


**Method Detail**

### computePrecision

```
public static double computePrecision(int tp,
                                      int fp)
```

:   Given counts of TPs and FPs, compute the precision

    :   **Parameters:**: `tp` - the number of true positives: `fp` - the number of false positives **Returns:**: TP/(TP+FP)

---


### computeRecall

```
public static double computeRecall(int tp,
                                   int fn)
```

:   Given counts of TPs and FNs, compute the recall

    :   **Parameters:**: `tp` - the number of true positives: `fn` - the number of false positives **Returns:**: TP/(TP+FN)

---


### computeFMeasure

```
public static double computeFMeasure(double precision,
                                     double recall)
```

:   Given precision and recall, compute the F-measure

    :   **Parameters:**: `precision` -: `recall` - **Returns:**: 2PR/(P+R)

---


### computeFMeasure

```
public static double computeFMeasure(int tp,
                                     int fp,
                                     int fn)
```

:   Given counts of TPs, FPs, and FNs, compute the F-measure

    :   **Parameters:**: `tp` -: `fp` -: `fn` - **Returns:**: 2PR/(P+R)

---


### computeClassicDice

```
public static double computeClassicDice(Set<String> aTokens,
                                        Set<String> bTokens)
```

:   Computes the Dice Coefficient as described in Hersh et al. TREC Genomics Track Overview, 2003.

    For two `Strings` A and B, define X as the number of words in A, Y as the number of words in B, and Z as the number of words
    occurring in both A and B. The Dice coefficient is calculated as Dice(A,B) = (2\*Z)/(X+Y)

    :   **Parameters:**: `aTokens` - a `Set` of tokens from `String` A: `bTokens` - a `Set` of tokens from `String` B **Returns:**: the Dice Coefficient for the two sets of tokens

---


### computeClassicDice

```
public static double computeClassicDice(String aString,
                                        String bString)
```

:   Computes the Dice Coefficient as described in Hersh et al. TREC Genomics Track Overview, 2003.

    For two `Strings` A and B, define X as the number of words in A, Y as the number of words in B, and Z as the number of words
    occurring in both A and B. The Dice coefficient is calculated as Dice(A,B) = (2\*Z)/(X+Y)

    This method tokenizes by splitting the input Strings at spaces.

    :   **Parameters:**: `aString` - a `String` to compare: `bString` - another `String` to compare **Returns:**: the Dice Coefficient for the two input Strings


---


|  |  |  |
| --- | --- | --- |
| **Overview** \| **Package** \| **Class** \| **Tree** \| **Deprecated** \| **Index** \| **Help** | |  |
| PREV CLASS   **NEXT CLASS** | **FRAMES**    **NO FRAMES**     **All Classes** |  |
| SUMMARY: NESTED \| FIELD \| CONSTR \| METHOD | DETAIL: FIELD \| CONSTR \| METHOD |  |


---
